# Supplementary material for: Are carbon emissions trading and green financial instruments synergistic? -Comprehensive quantitative research based on content analysis
Source: PLoS One. 2024 Mar 7;19(3):e0298601. doi: 10.1371/journal.pone.0298601 (PMC10919601; doi:10.1371/journal.pone.0298601)
Supplement: S1 Data — (ZIP) [file pone.0298601.s001.zip › Data/地级市Z_F工作报告.zip!/╣π╢1⁄2/╒╪╟∞╩╨/└┤╫╘╣1⁄2╓┌║┼í╛┬φ┐╦╩2╛▌═°í┐_2007.docx]

各位代表：

　　我代表肇庆市人民政府向大会作政府工作报告，请予审议，并请政协各位委员和其他列席人员提出意见。

　　一、本届政府工作回顾

　　本届政府在中共广东省委、省人民政府和中共肇庆市委的正确领导下，在市人民代表大会及其常委会和市政协的支持监督下，坚持以邓小平理论和“三个代表”重要思想为指导，全面落实科学发展观，按照市第九次党代会确定的目标任务和市委各个阶段的工作部署，坚持发展第一要务，抢抓机遇，求真务实，开拓创新，艰苦奋斗，团结带领全市人民不断开创工作新局面，推动经济持续快速健康发展，社会各项事业全面进步，精神文明和民主法制建设迈上新水平，实现了“一年打基础、三年见成效”的工作目标，完成或超额完成了市十届人大历次会议确定的任务，为实现“十一五”时期发展目标，建设繁荣活力、文明法治、和谐安康肇庆打下了坚实基础。

　　(一)综合经济实力不断增强，经济发展预期目标顺利实现

　　坚持以经济建设为中心，积极实施东进和“东引西连”发展战略，实行内、外源型经济并举，全力打造“三大经济板块”，推动经济增长不断创新高。2003年到2005年,肇庆市生产总值年增速分别为11.4%、13.2%、14.4%，2006年预计实现526.22亿元(绝对值按现行价，对比按不变价，下同)，增长14.5%，超过预期目标1.5个百分点，比2002年增长65.2%；地方财政一般预算收入实现24.98亿元，比上年增长22.2%，超过预期目标8.2个百分点，比2002年增长95.5%；全社会固定资产投资完成216.48亿元，比上年增长21.6%，比2002年增长121.9%，近四年投资总额达655亿元。全市呈现经济发展持续加快、三大产业同步发展、综合实力不断增强、人民生活逐步迈向富裕安康的良好局面。

　　工业持续快速发展。坚持以工业为主战场，实行引进项目与培育骨干企业相结合，近四年共引进投资1000万元以上工业项目525项，投资总额340多亿元，一批实力雄厚、关联带动能力强的大企业、大项目，如韩国SK、美国LP和亚洲铝业等先后落户我市，目前规模以上工业企业已有760户。不断调整优化产业结构，发展壮大了电子信息、生物制药、金属加工、汽车配件、建筑陶瓷、林浆纸一体化等支柱产业。国有企业改革不断扩大深化，市属中小国有工业企业大部分实施了产权改革，星湖科技和风华高科股权分置改革顺利完成，蓝带啤酒企业实行产权改革后扭亏为盈，市属国有企业整体经济效益提高。广东鸿图企业成为我市第3家A股上市公司。民营工业不断发展壮大，2006年，预计全市民营工业总产值实现234.2亿元，占全市工业总产值的45.7%，比上年增长35.5%。工业技术改造步伐加快，近四年工业技改投资29.56亿元，共有95个技术改造和技术创新项目列入国家和省的计划。2006年，预计全部工业增加值实现130.91亿元，比上年增长32.0%，比2002年增长125.5%；规模以上工业企业实现利税总额22.8亿元，比上年增长21.5%，规模以上工业增加值增幅排在全省地级市的前列。

　　招商引资不断取得新突破。把招商引资放在经济工作的龙头地位，积极创新招商方式和服务机制，成功举办四届“肇庆金秋”经贸洽谈会，不断掀起招商引资高潮。2003年以来累计实际吸收外资达24.3亿美元，其中2006年预计6.71亿美元，比上年增长11.4%，吸收外资总量连续四年稳居全省山区市首位。全力抓好招商引资载体建设，大力发展园区经济，已有966户企业落户各级工业园区。肇庆高新区被省政府确定为广东省吸收外资重点园区和山区吸收外资示范区，近四年累计实际吸收外资8.7亿美元，成为我市招商引资的排头兵。承接珠三角产业转移取得突破，近四年累计承接珠三角地区产业转移企业528户，合计投资322亿元。德庆县与顺德区龙江镇、怀集县与中山市大涌镇共建产业转移工业园已获省政府批准认定。

　　社会主义新农村建设加快推进。以促进农业发展、农民增收为重点，积极开展新农村建设，推进农村工业化、城镇化和农业产业化，促进了农业和农村经济稳定发展。农村工业化进程加快，资源型工业和特色工业不断发展壮大，建立起规模化生产的林浆纸、新型建材、林产化工和矿产冶炼等产业基地。县域经济发展提速，4个山区县规模以上工业增加值从2002年的7.27亿元增加到2006年的17.18亿元，年均增长24%，高于全市同期平均增长水平。农业产业化加快推进，建成了全省规模最大的柑桔种植基地，县级以上农业龙头企业发展到102家，有9个农产品被评为省级名牌产品、5个农产品通过绿色食品认证。粮食生产保持稳定，去年为帮助受灾群众恢复生产，紧急动用救灾种子20万斤，并火速从外地购入种子10万斤，全年粮食获得丰收。2006年，预计农业总产值实现215.74亿元，比上年增长5.8%，超过预期目标0.3个百分点，比2002年增长19%。城镇化水平不断提高，中心镇总体规划修编工作全面完成，加快农村基础设施建设和农村公用事业发展，村镇建设、管理得到加强。积极开展生态文明村建设并得到省的肯定，累计创建生态文明村1155条，其中2006年创建450条，农村生态环境有较大改善。

　　第三产业稳步发展。完成编制和启动旅游发展总体规划，实施旅游管理体制改革，积极引入旅游大企业参与旅游资源开发和旅游产品推介。提升星湖风景名胜区品位，打造县域特色旅游品牌，形成千里旅游走廊并成为广东省旅游热线。旅游配套设施建设加快，第一家按五星级标准设计的酒店已基本建成，正在建设的4家；2006年全市旅游景区接待旅游者首次突破1000万人次，仅次于广州、深圳，成为全省第三大旅游景区接待城市，预计旅游收入达到47.46亿元。出台了扶持物流业发展的政策措施，引入物流企业参与港口资源整合开发。一批规模大、档次高的商贸超市相继开业，2006年预计社会消费品零售总额167.9亿元，比上年增长18%，比2002年增长74.6%。房地产发展水平不断提高，中高档房地产稳步发展，预计房地产投资25.04亿元。外贸出口保持增长，预计外贸出口总额17.6亿美元，比上年增长23%。

　　重大项目谋划建设有效推进。积极实施重大项目带动战略，一批对我市经济发展起带动促进作用的重大项目得到落实。亚洲铝业首期工程、德庆石井水泥首期工程等已建成投产，亚洲金属资源再生工业基地等一批工业项目正抓紧规划建设。西江大桥扩建工程、肇庆大桥南引道改建工程竣工通车，二广高速公路三水至广宁段全面动工建设。国道321线文明样板路先行开工路段改造已经完成，其余路段全线动工。珠三角外环高速公路肇庆段、广肇高速公路二期工程正在开展前期工作。西江航道肇庆至虎跳门3000吨级航道整治工程顺利完成，肇庆至封开界首2000吨级航道整治工程、肇庆新港和绥江牛岐船闸改建工程建设加快推进。景丰联围加固达标工程已上报国家发改委审批。谋划内河核电取得阶段性成效，肇庆电厂项目列入全省“十一五”能源建设规划，大旺铝西110千伏、高要砚都500千伏输变电工程建成使用。

　　人民生活水平明显提高。经济发展惠及人民，城乡居民生活得到较大改善。2006年，预计城镇居民人均可支配收入11460元，比上年增长13.5%，比2002年增长56.2%；农村居民人均纯收入4776元，比上年增长4.8%，比2002年增长20.4%；年末城乡居民储蓄存款余额380.5亿元，比年初增长13.2%，比2002年增长69.2%。

　(二)社会各项事业全面进步，构建和谐肇庆取得实效

　　科教兴市迈出新步。科技服务体系不断完善，科技创新水平不断提高，近四年累计实施各类科技计划325项，获国家级科技进步奖2项、省级科技进步奖38项，建成省级以上工程技术研发中心11家，拥有各级高新技术企业112户、民营科技企业99户、专业镇11个、国家免检产品6个、广东省工业名牌产品26个、广东省工业著名商标17个。信息化加快推进，被确定为国家电子信息产业基地和全省山区信息化示范市。建成鼎湖山院士活动基地。通过了2003－2004年度全国科技进步考核，被评为全省科技进步先进城市。与中山大学建立了校市全面合作关系。加强人才引进和干部培训，先后组织各级领导干部983人次到中山大学短期培训，选送58名年轻干部赴英国进修学习，参加市委党校以上干部脱产培训5537人。教育事业蓬勃发展。累计改造农村和老区、山区中小学校504所，中小学布局调整工作成为全省先进典型。全面落实农村义务教育阶段免收杂费政策，近四年城区新增基础教育学位7390个，“普九”各项指标达到或超过国家考核要求。成功举办第二届教育百万行活动。幼儿教育、普通高中、职业教育发展加快。万人口在园幼儿人数居全省第四位；近四年新增高中优质学位2.5万多个；建立了全省首个职业技术教育培训基地，职业教育发展速度和规模居全省地级市第3位，成功举办职教基地发展论坛暨职教合作项目签约仪式，签订投资金额达5亿多元的办学项目。高等教育快速协调发展，新增高等院校4所，高等教育毛入学率提高到20%。

　　城市规划建设不断加快。编制完成《肇庆市概念性总体规划》，开展“山、湖、城、江”城市特色研究，完成了“肇庆府城”保护与复兴、肇庆市近期建设等多个专项规划。近四年市本级组织投入中心城区建设资金达9.65亿元，完成了宋城路、黄塘路等道路的改造升级，实施了城区“美化、绿化、亮化”工程，城区面貌焕然一新。城东新区基础设施建设项目加快推进，首期工程可在今年交付使用。我市被评为中国投资环境百佳城市。

　　生态环境保护卓有成效。高度重视生态环境保护，全面启动治污保洁和西江综合整治工程，投入3800万元整治星湖水环境，新增城市污水处理厂1座，城区生活污水处理率提高到71%，全市地表水和饮用水源水质100%达标。有6家企业获得广东省清洁生产先进企业称号。农林、矿产资源管理得到加强。造林绿化实现“三年灭荒”目标，全市森林覆盖率提高到65.1%，广宁县被授予全国绿化模范县称号，封开县获得国家地质公园称号。新建了3个省级、5个市级自然保护区和肇庆星湖湿地公园。我市被评为国家环境保护模范城市。

　　社会事业全面发展。全面建设疾病控制、医疗救治和卫生监督三大体系，加强疫情报告和突发公共卫生应急处理，积极防治艾滋病，成功抵御“非典”、人禽流感等重大传染病疫情。出色完成医疗援外任务。建成“广东省卫生村”100条。通过国家卫生城市复查。人口和计划生育工作进一步加强，近四年人口出生率和自然增长率均低于省下达的目标要求。全民健身活动蓬勃开展，竞技体育屡创佳绩，我市体育健儿在第十二届省运会夺得金牌103枚，名列全省第四名，连年荣获“广东省体育贡献奖”。全民国防教育深入开展，国防后备力量、国防动员和人民防空建设有新进展，连续四届获得全省“双拥模范城”称号。驻肇部队和民兵预备役在承担抢险救灾等急难任务中发挥了突击队作用。殡葬管理目标考核年年达标。镇村撤并(改制)工作超额完成省下达的目标任务。社会中介和民间组织管理工作不断加强，社会慈善事业加快发展。外事、侨务工作得到新发展，与英国波尔顿市结为友好城市。妇女儿童、民族、宗教、地方志、新闻出版、气象、档案、老龄、残疾人等工作不断取得新成绩。

　　精神文明建设深入推进。深入开展“三个代表”重要思想和科学发展观学习实践活动，掀起学习《江泽民文选》热潮。组织“爱国、守法、诚信、知礼”现代公民教育活动，大力宣传贾东亮艰苦创业精神和马红梅、王继贤抗洪救灾英雄先进事迹。文化名市建设扎实推进，文化事业和文化产业同步发展。修复了白沙龙母庙、德庆学宫、宋城墙等历史文物，挖掘改造一批集生态、文化、旅游于一体的古村落，完成50户以上村村通广播电视。荣获“中国砚都”称号，端砚文化村建成开放。成功承办国家历史文化名城2005年年会。《端砚制作技艺》和《悦城龙母诞庙会》分别入选第一批国家级和省级非物质文化遗产名录。“学习型城市”加快构建，组织多期“星湖讲坛”，举办了首届“读书节”。

　　民主法制建设得到强化。积极推进依法治市，严格落实向人民代表大会及其常委会报告工作、向人民政协通报情况制度，认真办理各类建议、议案、提案。2003年以来，累计向市人大常委会提交专项工作报告59件，组织办理人大代表建议465件，政协提案、建议557件。贯彻落实《行政许可法》，有效促进依法行政。重大事项社会公示和听证制度进一步落实。积极维护司法公正与社会公平正义。重视听取民主党派、无党派人士和人民团体的意见建议。“四五”普法工作被省评为“优秀”，“五五”普法工作全面启动。基层民主法治建设深入推进，创建“民主法治示范村”695条。

　　社会大局保持稳定。加强社会治安综合治理，治安防控体系逐步完善，严厉打击各种违法犯罪行为，建立了“预防、排查、包案、处置、联动、督办”等预防、处置社会矛盾和集体上访六项工作机制。组建平安和谐社区示范点10个，创建安全文明小区1990个。信访工作机制不断完善，矛盾纠纷排查调处工作成效突出，群体性事件连年下降。被评为“全国社会治安综合治理优秀地市”、“全省维护稳定及社会治安综合治理先进市”。加强了食品药品监督检查，“打假”责任制得到落实。安全生产形势总体保持平稳。

　　民心工程得到有效落实。坚持执政为民，认真解决群众生产生活困难问题。就业和再就业工作进一步加强，近四年城镇新增就业岗位12.34万个，城镇登记失业率均控制在2.9%以内，转移农村富余劳动力就业23.42万人次。社会保险参保人数和基金收入不断增加，征缴率保持在98%左右。社会保险待遇支付率保持100%。出台实施优化外来人员务工环境的政策措施。扩大了法律援助社会覆盖面。安居工程建设加快推进，肇庆城区首批经济适用房和廉租房已向住房困难户公开发售和出租。社会救助体系不断完善，实现城乡低保动态管理下的“应保尽保”。全面落实免征农业税等支农惠农政策。严格执行建设项目征用地政策，全面兑现征用农民集体土地应补偿金额。认真解决农村“一保五难”(即最低生活保障和行路难、饮水难、读书难、看病难、住房难)问题，对农村近20万人实施低保救助，已有近3万农村人口脱贫；共55.8万人次享受了免收义务教育阶段书杂费政策；完成危房改造8802户，受益农民约4万人；完成镇通行政村公路改造2356公里，占总任务的93.7%；解决了18.3万农民饮水难问题；累计投入2986万元改造乡镇薄弱卫生院；去年新型农村合作医疗覆盖率达到52.8%。积极帮助灾区恢复生产、重建家园。去年5月至8月，我市连续遭受“珍珠”、“碧利斯”、“派比安”等台风洪涝严重自然灾害袭击，在省委、省政府的关怀和市委的正确领导下，全市党政军民万众一心，全力抗洪救灾，确保了人民群众生命财产安全，确保了把损失降到最低限度，使受灾群众人人有饭吃、有水喝、有衣穿、有地方住、有病能及时得到治疗，灾后又多渠道筹措资金1.5亿元,帮助灾民恢复生产、重建家园，全市4404户全倒户新居已在元旦前全部建成。

　　(三)政府管理体制得到创新，机关作风不断改进

　　行政管理机制逐步完善。明确建设为民、高效、廉洁、务实、有为政府目标，建立健全政府工作制度和决策规则，促进了行政行为科学化、法制化、规范化。落实了政府工作目标责任制。开展了三轮行政审批制度改革，共取消449项行政审批许可项目。积极简政放权，把80多项市级审批事项和管理权限下放到县(市)区，将原属肇庆高新区的三榕工业园移交端州区管理。加快体制创新步伐，初步建立国有资产监管营运体系，逐步开展政府公共资源配置市场化运作和实行收益集中管理。不断深化市属国有企业改革，实施改革转制企业255家，筹措改革资金6.63亿元，安置职工22387人。建立了层级应急管理机构和突发公共事件应急处置机制。成立政府行政服务中心，建立了“一站式”电子政务服务系统和行政审批电子监察系统。国税、地税部门，海关、海事及各口岸联检单位，工商管理、质量监督、食品药品监督等中央、省驻肇单位和金融机构不断创新工作方法，为支持地方经济发展作出了积极贡献。

　　勤政廉政建设进一步加强。实行市直机关公务员统一发放岗位津贴制度。开展了国库集中支付改革试点和财政支出绩效评价试点。全面清理市直党政机关、事业单位经营性资产并建立完善监管制度。纠正违法征用土地、清欠农民工工资、查处企业违法排污、减轻农民负担、治理教育乱收费、治理公路“三乱”、治理商业贿赂等专项工作取得明显成效。行政效能监察力度加大，开展了政府决定事项的专项督查。全面实行政务、厂务、村务公开，市、县两级政府门户信息网站基本建立。落实政府廉政工作责任制和各项审计制度，严肃查处违法违纪行为。认真开展机关作风和民主评议政风行风活动，在媒体开辟了“行风热线”栏目，促进了机关作风的转变。

　　回顾本届政府三年多来的工作，我们深刻体会到，要实现加快发展、跨越发展、科学发展，加快构建和谐肇庆，必须切实做到“六个坚持”：一是必须坚持树立和落实科学发展观，以经济建设为中心，统筹经济社会和城乡区域协调发展，不断壮大经济规模，提高发展质量，为构建和谐社会奠定物质基础。二是必须坚持把工业放在发展经济的主导战略地位，大力开展招商引资。突出工业主战场，优化提升产业结构，加快工业规模化、集约化，推动工业经济率先实现跨越发展。以招商引资为手段，积极承接珠三角产业转移，加强“泛珠三角”区域合作，发展壮大外资、民营经济。三是必须坚持重大项目带动战略，把谋划建设重大项目作为实现跨越发展的重要支撑，进一步增强经济发展后劲。四是必须坚持实施可持续发展，加强资源综合利用，大力发展循环经济，强化生态环境保护建设，构建资源节约型和环境友好型社会。五是必须坚持体制机制创新，深化经济体制改革，增强自主创新能力，提高经济增长质量和效益。六是必须坚持执政为民，认真解决群众生产生活困难，努力提高人民生活水平，让人民群众共享发展成果。

　　各位代表，本届政府工作目标任务的顺利完成，是省委、省政府和市委正确领导的结果，是市人民代表大会及其常委会和市政协支持监督的结果，是全市人民实践“三个代表”重要思想，树立和落实科学发展观，解放思想、开拓进取、艰苦奋斗、心齐实干的结果，也是各民主党派、人民团体、无党派人士和社会各界关心支持的结果。在此，我代表肇庆市人民政府，向全市工人、农民、干部、知识分子、外来务工人员、驻肇单位、驻肇部队、武警官兵以及各界人士致以崇高的敬意！向关心和支持我市发展的港澳台同胞、海外侨胞和国际友人表示衷心的感谢！

　　在充分肯定成绩的同时，我们必须清醒地认识到，我们的工作离上级的要求和人民群众的期望还有较大差距，我市经济社会发展仍然存在不少困难和问题：一是经济总量不大、经济实力不强、区域发展不平衡。二是产业结构不够优化，龙头企业和支柱产业不明显，自主创新能力不够强。三是体制性障碍仍然存在，社会管理体系不够完善。四是部分重大项目资金到位率不高，一些招商引资项目未能够按计划落实。五是人民群众物质文化生活水平有待提高，社会保障水平还比较低，在教育、医疗、就业等事关民生方面的问题仍不少。六是政府工作作风建设仍需加强，部分机关办事效率仍较低，工作人员的服务意识和服务水平有待进一步提高。对此，我们必须高度重视，采取切实有效的措施认真解决。

二、新一届政府的工作目标和主要任务

　　未来五年，我市经济社会发展机遇与挑战并存，希望和困难同在。世界经济进入相对稳定的增长周期，产业结构调整和向外扩张转移不断加大。国家确立了科学发展的执政理念与和谐社会的建设目标，宏观经济环境继续向好。我省实施“泛珠三角”发展战略和“粤东西北振兴计划”，推动珠三角发达地区产业转移扩张，有利于促进我市与不同类型地区的互动协调发展。经过多年的努力，我市基础设施不断完善，发展环境不断优化，发展后劲不断增强。特别是近几年来，全市上下形成心齐实干的良好氛围，积累了发展经验，我市具备了进一步加快发展的良好环境和条件。但是，我市经济发展水平与珠三角核心地区相比仍有不少差距，区域竞争日趋激烈，我们面临的困难仍然很多。我们要继往开来，坚定信心，加油鼓劲，乘势而上，进一步增强工作的紧迫感和责任感，按照“继承、创新、发展”的要求，统一思想、把握机遇、明确任务、扎实工作，推动经济社会加快发展、跨越发展、科学发展，努力完成市第十次党代会确定的目标任务和“十一五”发展规划。

　　新一届政府的工作要求和总体目标是：以邓小平理论、“三个代表”重要思想和科学发展观统领经济社会发展全局，认真落实党的十六届六中全会、省委九届九次全会精神，按照市第十次党代会的决策部署，狠抓发展第一要务与和谐社会建设，坚持东进和“东引西连”战略取向，按照“三大经济板块”的总体布局，创新发展思路，突出重点，狠抓落实，实施“工业主导、重大项目带动、三产旺市、科教兴市和人才强市、区域协调发展”五大战略，促进经济又好又快发展，努力建设繁荣活力、文明法治、和谐安康、生态环保肇庆。到2011年，全面实现“十一五”规划任务，工业化、城镇化和农业产业化明显加快，实现地区生产总值年均增长13%以上，经济总量比2006年接近翻一番，地方财政一般预算收入年均增长13.5%，万元GDP能耗平均每年下降3.43%以上，经济实力明显增强，发展质量明显提高，综合竞争力明显提升；民主法制更加健全，行政效能进一步提高，社会管理不断完善，社会稳定基础进一步夯实；文化名市建设扎实推进,和谐社会建设明显进步，实现创建全国文明城市目标，惠及全民的公共服务体系基本建立，社会事业全面发展，社会保障体系逐步完善，社会就业更加充分，收入分配更加合理，城乡居民收入有较大提高；生态环保建设水平明显提升，“三个最适宜”城市建设目标基本实现，资源节约型和环境友好型社会初步建立；繁荣活力、文明法治、和谐安康、生态环保肇庆建设取得明显成效，与全省同步全面建成小康社会，率先基本实现社会主义现代化。

　　新一届政府的主要工作任务：一是坚定不移抓好发展第一要务。以科学发展观统领经济社会发展全局,坚持东进和“东引西连”战略取向,推进三大经济板块建设，加速发展工业、城市、县域、港口物流经济，壮大经济总量，提高发展质量，增强经济实力和综合竞争力。在推动经济加快发展的同时，更加注重环境保护，发展循环经济，积极构建资源节约型和环境友好型社会，再创发展新局面。二是坚定不移抓好工业、招商引资和重大项目建设。以工业为主战场，坚持走新型工业化道路，优化工业产业布局，实行引进项目和培植壮大骨干企业并重。加快发展产业集群，培育龙头企业，打造支柱产业，大力发展创税型、科技型、生态型企业，以工业的跨越发展带动全市经济的跨越发展。优化招商引资环境，提高招商引资质量，着力打造一批品牌园区和产业转移基地，加速东南部地区与珠三角核心区的产业对接，力争成为珠三角世界制造业基地的重要组成部分，做大做强内、外源型经济。继续谋划重大项目，抓好“十一五”时期安排的137项总投资约700亿元的工业、交通、能源和城市重大项目建设，增强经济发展后劲。三是坚定不移抓好社会主义新农村建设。按照“生产发展、生活宽裕、乡风文明、村容整洁、管理民主”的要求，努力建设有肇庆特色的社会主义新农村。贯彻工业反哺农业、城市支持农村和多予少取放活的方针，加快发展农村经济，增加农民收入，推进农村工业化、城镇化和农业产业化，发展壮大县域经济，扶持壮大特色产业和资源型工业，强化农村基础设施建设，发展农村公共事业，抓好中心镇、专业镇和生态文明村规划建设，促进城乡和区域协调发展。四是坚定不移抓好城市规划建设管理。围绕“三个最适宜”城市建设目标，加快发展以旅游为龙头的第三产业，搞活搞旺城市经济。做好中心城区规划修编，加快桥梁、道路等基础设施建设，畅通鼎湖、高要与端州对接发展，实现中心城区“东扩南连”、“东西呼应”、“南北互动”，努力构建“山、湖、城、江”特色要素和谐相融、一江两岸、超百万人口的区域中心大城市。加强生态环境保护，进一步提高城市“美化、绿化、亮化”水平。五是坚定不移抓好创新发展。推进科技体制改革，加快信息化步伐，完善以企业为主体的自主创新体系，提升自主创新能力。深化行政体制改革，促进依法行政。加强国有资产监管营运体系建设。加快构建公共财政管理体制，增强供给公共产品和公共服务的能力。推进要素市场建设，完善社会主义市场经济体制。六是坚定不移抓好和谐社会建设。坚持以人为本，努力实施技能培训工程、教育均衡发展工程、便民廉医工程、全民安居工程和生产生活环境改造工程等“五大民生工程”。完善利益分配机制，提高低收入者收入水平，认真解决群众生产生活困难；落实统筹城乡就业政策，扩大社会就业和再就业；健全社会保障安全网，加大对低收入群体的救助力度。优先发展教育，促进义务教育均衡化、高中教育普及化和高等教育大众化。加快文化名市建设，促进文化繁荣。完善公共卫生体系建设，提供公平基本医疗服务。推进民主法制建设，加强社会治安综合治理；加强公共安全管理，确保社会安定和谐。

三、2007年政府工作安排和措施

　　2007年是新一届政府工作的开局之年，我们必须按照科学发展观的要求，全面贯彻落实市第十次党代会精神，进一步解放思想、转变作风，全力推动经济发展，加快构建和谐社会，努力实现速度、质量、效益相协调，消费、投资、出口相协调，人口、资源、环境相协调，确保各项目标任务的完成。今年经济社会发展预期目标是：肇庆市生产总值增长14%，万元GDP能源消耗降低3.43%，全部工业总产值增长25%，农业总产值增长5%，全社会固定资产投资增长20%，社会消费品零售总额增长15%，外贸出口增长15%，实际吸收外资增长10%，地方财政一般预算收入增长15%，城镇登记失业率控制在3%以下，人口自然增长率控制在6.7‰以下，城镇居民人均可支配收入增长8%，农村居民人均纯收入增长5%。重点要抓好以下工作：

　　(一)加快壮大工业总量，提高工业发展质量。工业是我市能否实现跨越发展的关键所在，必须强化工业主导地位，优先发展，加速发展，走新型工业化道路。要继续调整优化工业结构，发展壮大支柱产业，积极发展装备制造业，扶持壮大电子信息、饮料食品、生物制药、金属加工制造、汽车配件、林浆纸一体化、新型建材等有较强竞争优势的产业。抓好产业基地发展，优化产业布局，延伸产业链，促进集约建设、集群发展，提升工业核心竞争力。扶持壮大龙头骨干企业，提高国有重点企业可持续发展能力，支持民营、外资骨干企业做大、做强、做优。加强企业技改扩产的支持力度，重点扶持带动效应大、创税能力强的重大技改项目。加快发展信息产业，以信息化带动工业化。增强企业科研水平，培植壮大名优名牌产品，巩固提升“肇庆制造”，着力打造“肇庆创造”。积极发展清洁能源和可再生能源，降低单位生产总值能耗比，培育发展科技含量高、经济效益好、能源消耗低、环境污染少、人力资源优势得到充分发挥的新型工业项目。

　　(二)全力抓好招商引资，加快谋划建设重大项目。招商引资和重大项目建设是迅速壮大经济总量，增强经济发展后劲的最根本、最有效途径。要加大招商引资工作力度，落实招商引资工作责任制，创新招商引资方式，扩大“肇庆金秋”品牌效应，切实提高项目履约率。鼓励支持现有外资企业增资扩产，大力推行中介招商、以商引商，积极实施园区招商和产业招商，实行资源型和非资源型项目引进并举，严格控制污染、高危项目，注重引进发展投资规模大、技术含量高、产业链条长、创税能力强的项目，力争招商引资有新突破。强化招商引资载体建设，创新完善肇庆高新区管理体制，提升园区综合环境水平，打造主导产业集群，为申报国家级高新区打好基础。积极谋划建设肇庆(高要)省级工业园。大力支持各县(市)与珠三角核心地区对接建立产业转移园区。落实推动民营经济发展的政策措施，放手、放心、放胆发展民营经济，完善社会化服务体系，创新中小企业融资方式，促进民营经济加快发展。加大力度谋划建设重大项目，推动在建项目早日建成、签约项目尽快动工、在谈项目加紧落实。抓好亚洲铝业二期、鼎丰纸业技改扩产、亚洲金属资源再生工业基地等项目的建设。加快二广高速公路三水至怀集段建设，推进广肇高速公路二期工程，基本完成国道321线高要小湘至封开涌口段文明样板路改造工程，力争肇庆新港首期工程投产，配合做好珠三角外环高速公路肇庆段、西江航道肇庆至封开界首段整治工程、封开大桥和广梧高速公路封开连接线等项目的前期准备工作并争取年内动工建设，配合中国广东核电集团和有关机构开展西江核电项目可行性研究工作，力争肇庆电厂一期工程通过国家核准。加快景丰联围、大旺围加固工程建设速度，推进中心城区城东新区基础设施项目建设，力争主干道年内建成。

　　(三)扎实推进社会主义新农村建设，发展壮大县域经济。全面建设小康社会，关键在农村，难点也在农村，必须认真贯彻落实中央和省的决策部署，以发展农村经济为重点，扎实推进社会主义新农村建设。要把解决好“三农”问题放在重中之重，着力发展现代农业，提高农业综合生产能力，促进农村经济发展、农民增收。加快农业结构调整，发展品牌农业、高效农业、特色农业，稳定粮食生产，扩大优质畜禽水产养殖、中华名优果品、高效林业及特色林产品、绿色食品、南药的生产。大力扶持农业龙头企业和农民专业合作组织，加强农产品流通基地和标准化生产基地建设，完善农业社会化服务体系。积极引进和发展资源深加工型、劳动密集型和传统优势型产业，大办特色工业，做大做强县域工业经济。推进农村基础设施建设，积极实施防灾减灾工程，完善水利设施。全面完成镇通行政村道路硬底化建设，提高行政村客运班车通达率。加快农村电网改造升级，实现全市用电同网同价。完善动植物防疫网络，防止发生重大动植物疫情，建成县、镇、村三级自然灾害监测网。加快中心镇规划和建设，抓紧编制和实施村庄规划。继续抓好“千村生态文明工程”，力争新建500条生态文明村。

　　(四)加快旅游业发展，提高第三产业发展水平。旅游、外贸、物流、房地产等第三产业，对促进经济繁荣兴旺具有十分重要的作用。必须发挥资源优势，加大旅游体制改革和旅游业开放力度，支持各类社会资本参与旅游景区开发经营。加强旅游开发的规划引导，以中心城区旅游为龙头，以千里旅游走廊为载体，加快旅游资源与文化资源的整合，巩固发展观光旅游，大力培育休闲、度假、会议、商务和乡村旅游，打造一批富有历史文化内涵、参与性强的旅游精品项目。加大省外、境外宣传推介力度，打造中国最佳旅游线路广东必游热线。抓好住宿、购物、娱乐等旅游配套项目建设，扶持发展一批中高档酒店和休闲购物娱乐场所。围绕建设西江流域物流枢纽城市目标，优化港口布局，整合港口资源，抓好港口物流园区规划建设，积极谋划工业、商业、保税物流园区，加快发展港口物流经济。正确处理好投资和消费、内需和外需的关系，努力扩大消费需求。大力拓展多元化外贸出口，支持外贸出口企业优化产品结构，打造出口品牌。继续加强投资软硬环境建设，优化“大通关”环境，提高口岸通关效率。优化房地产业发展结构，提高住宅小区开发建设水平，加快发展普通商品住宅。鼓励传统商贸服务业转型升级，积极拓展农村消费市场，完善农村商贸服务网点，促进城乡扩大消费。大力发展金融、保险、会展、商务、咨询、医疗等现代服务业。

　　(五)着力抓好市直经济，开创市直经济发展新局面。发展壮大市直经济，不仅可以增强市本级的支配能力，而且有利于推动全市经济的发展。必须加大对市直经济统筹推进的力度。成立市直经济工作领导小组，建立市领导和市经济部门联系重点企业制度，强化工作责任和考核。认真实施《关于加快发展市直经济的若干政策措施》，优化市直经济的发展环境。把招商引资作为加快市直经济发展的重要途径，突出抓好高新区和市城区两个主战场的招商引资工作，着力培植市直工业龙头企业和支柱产业，重点扶持以风华高科为龙头的电子信息产业、以蓝带啤酒为龙头的食品饮料产业、以星湖科技为龙头的生物制药产业、以本田金属为龙头的汽配产业、以亚洲铝业为龙头的金属加工产业等五大支柱产业。加快发展城市经济，重点发展以旅游、房地产和商贸物流为重点的第三产业。

　　(六)切实做好财税工作，努力改善金融服务环境。财税是地方综合经济实力的重要体现，关系到政府的调控能力和执政能力。必须积极培植税源，提高财政增长质量。推进公共资源合理配置和特定行业经营市场化运作，强化国有资产收益集中统筹管理，严格执行非税收入监管制度，努力拓宽财政增收渠道。支持税务部门加快“金税工程”升级换代，提高税收征管水平，严格依法治税。推进公共财政管理体制建设，优化财政支出结构，重点保障义务教育、公共卫生、社会保障、社会就业和环境保护等公共领域的支出，做好公务员工资改革。加强财政支出监管和部门预算管理，深化国库集中支付改革，积极实施财政支出绩效评价制度。加强政府债务管理，建立政府债务风险评价预警体系。创造良好的金融环境，协调金融机构防范金融风险，坚决打击恶意逃废银行债务行为。深化银企合作，完善担保体系，支持金融机构创新运行机制和服务手段，加大对地方经济社会发展的支持。

　　(七)推进体制创新和科技创新，完善市场经济体系。创新是实现经济社会快速协调发展的保证和支撑，必须进一步抓好体制创新和科技创新。要突出抓好市属国有企业的创新发展，推动重点企业改革重组，基本完成市属国有中小企业产权改革。切实维护转制企业职工合法权益，妥善解决历史遗留问题。全面落实国有企业预算管理、投资管理、绩效评价、责任审计等监管制度，健全国有企业经营者激励约束机制。深化投资体制改革，完善投资审批、核准和备案制度，严格实行政府投资项目预算约束制和绩效评价制，推行公益性项目代建制。推进社会信用体系建设，落实“打假”工作责任制，整顿规范市场经济秩序，加快发展社会中介服务组织。深化农村综合改革，探索建立农村集体土地承包经营权流转新机制。把提高企业自主创新能力作为科技工作的中心环节，继续扶持建设企业工程技术开发中心、技术创新专业镇和农业科技创新中心，加快在肇庆高新区建设省级产学研基地、创业服务中心、国家级金属新材料特色产业基地和中巴软件园大旺基地。开展科技示范镇(基地)试点，启动以四个山区县为主体的南药产业带、以绥江流域为主的竹业产业带和以高要为中心的水产产业带等星火技术产业带建设。深化与中山大学校市合作。继续实施高层次科技人才“211”工程，营造有利于人才干事创业的环境。完成市科技中心建设。加强知识产权和专利保护。

　　(八)加强城市规划建设管理，做好资源环境保护工作。城市规划建设水平，直接影响到我市形象和投资环境。要加快城镇规划建设管理，推进城市化进程，提升城市品位。完成修编《肇庆市城市总体规划(2006－2025)》，加强一江两岸统一规划，做好筹建西江三桥前期工作，加快中心城区东扩南连。继续实施城区道路改造升级，完成端州七、八路改造、宋城西路二期、星湖治污和西排南段改造等工程，实施开通人民北路工程，启动西江路、江滨堤路、星荷路改造工程。完成宋城墙及其周边环境的修建整治，新建东调洪湖和梅庵岗公园。大力实施环境保护规划，注重节能降耗、保护环境，落实层级环保实绩考核责任制和责任追究制，加强分类指导和分区控制，实现排污总量控制目标。加强环保基础设施建设，加强城乡环境综合治理，抓紧筹建市城区第三污水处理厂、肇庆高新区污水处理厂，力争全市城镇生活污水处理率提高到60%以上。加强星湖名胜区、鼎湖山自然保护区建设和管理，抓好九龙湖第二饮用水源地规划和保护。健全环保监督执法和监测预警体系，严厉查处环境违法行为，严格控制污水、废气、固体废弃物和噪声等工业污染。加强土地管理，提高土地利用率。严格落实征地项目“三条红线”制度，认真执行征地补偿保护标准。深入开展林业生态县、生态示范区、绿色社区创建活动，力争森林覆盖率稳定在66%以上，城市人均公共绿地面积增加到8平方米。

　　(九)继续推进教育强市和文化名市建设，全面发展社会各项事业。继续把教育摆在优先发展的战略地位，为经济社会发展提供强有力的智力支持和人才保证。加大教育投入，推进“双高普九”，着力解决端州城区基础教育学位紧缺问题。加快普通高中扩建扩招，继续做好国家级示范高中创建工作，抓紧建设肇庆中学新疆班，确保秋季正式招生。加快职教基地建设，推进职教园区和市实训中心建设，大力发展民办职业教育，理顺职教管理体制，把5所市属中职学校归口市教育行政部门管理。加快发展高等教育，支持肇庆学院完善办学条件，加快肇庆医专、科技学院、工商学院、技师学院新校区建设。推进“强师振教”工程，加大教师培训和引进力度，全面推进素质教育，力争全市教育综合实力再上新水平。深化文化体制改革，加强文化基础设施和公共文化体系建设，积极鼓励社会资本投资兴办文化基础设施和文化产业，挖掘和保护文化遗产，打造肇庆特色文化品牌。加强文化市场综合管理。抓好社会主义精神文明建设，以创建全国文明城市为目标，积极开展“八荣八耻”社会主义荣辱观和“爱国、守法、诚信、知礼”现代公民教育活动，广泛开展文明行业、文明村镇、文明单位、文明家庭等群众性文明创建活动。开展全民健身活动，发展竞技体育，办好市第八届运动会。大力发展卫生事业，重点发展公共卫生服务和农村卫生事业。深化医疗卫生体制改革，加强医疗服务市场监管，合理配置卫生资源。完善疾病控制、医疗救治和卫生监督体系建设，抓好重点传染性疾病预防控制。加快发展中医药，提高中医服务水平。进一步推进卫生村创建工作，新增省卫生村40条以上。稳定人口低生育水平，有效治理出生人口性别比升高问题，确保完成人口与计划生育工作目标。继续实施妇女儿童发展规划，切实保障老龄人和残疾人的合法权益。加强国防动员、民兵预备役和人防建设，巩固发展军政军民关系，争创全国“双拥模范城”。巩固和深化殡葬改革。落实民族和宗教政策。精心组织农业普查。做好外事、侨务、档案、地方志等工作。

　　(十)高度重视民生民利，积极实施“五大民生工程”。我们的宗旨是执政为民，必须坚持发展为了人民，发展依靠人民，发展成果由人民共享。积极推进技能培训工程，落实好农民工技能提升计划和下岗失业人员再就业培训计划，着力抓好农村青年、退役士兵、城市失业人员等群体职业技能培训，提高劳动者技能素质。努力构建城乡一体化的劳动力市场，加快建设农村劳动力综合培训基地。积极推进教育均衡发展工程，切实改善农村义务教育办学条件，市财政安排200万元全面推进农村中小学C、D级危房改造，着力解决好农村中小学教学生活设施等问题，使农村义务教育学校仪器设备基本达到部颁标准。扎实推进农村义务教育经费保障机制改革，在继续落实农村义务教育阶段免收杂费的基础上，从今年秋季开始免收课本费。开展“智力扶贫”，多渠道筹集经费建立助学金，解决城乡困难群众子女读书难问题。积极推进便民廉医工程，加强社区卫生服务，完善社区卫生服务网络。市财政安排200万元投入乡镇卫生院和卫生站建设。完善新型农村合作医疗制度，力争覆盖率达到75%以上，住院补偿提高到1万元以上。努力解决群众看病难、看病贵问题，认真落实对特困群众的各项医疗帮扶措施，努力为群众提供便捷、廉价、优质的基本医疗服务。建立和完善医疗救助制度，多渠道筹集救助资金。积极推进全民安居工程，今年市安排建设城区经济适用房5.84万平方米，解决城镇低收入家庭住房难问题，全面完成农村贫困户危房改造任务，有计划改造农村泥砖住房。积极推进生产生活环境改造工程，大力推广农村使用沼气，加快实现20户以上自然村通电话、通广播电视。抓好与人民群众生产生活密切相关的水、电、路、灯等设施改造。扩大社会保险覆盖面，完善社会保险市级统筹，探索实施适合农民工特点的社会保险办法，提高社会保险基金保障水平和抗风险能力。健全企业退休人员社会化管理服务网络。落实城乡居民的最低生活保障制度，做好救济工作。切实解决城镇特困户和农村五保户的基本生活问题。加大统筹城乡就业的工作力度，实施积极的就业政策，增加就业岗位，努力解决“4050”人员、领取城市最低生活保障的登记失业人员、“零就业家庭”成员的就业困难，帮助农村贫困家庭人员和被征地农民实现就业。完善工资协商和调解机制?落实最低工资制度，加大劳动保障监察力度，完善劳动争议仲裁机制，积极发挥工会组织的作用，维护职工合法权益，努力构建和谐劳资关系。

　　(十一)加强民主法制建设，切实维护社会大局稳定。加强社会主义民主法制建设，加快依法治市步伐，是促进我市经济发展、社会进步和社会稳定的重要保证。要认真落实市人大及其常委会的决议、决定，及时向人大及其常委会报告工作，接受其监督。大力支持人民政协履行政治协商、民主监督、参政议政职能。认真听取民主党派、工商联、无党派人士和人民团体的意见。办好人大代表意见、建议和政协提案。认真做好行政复议和行政应诉工作。严格执行听证制度和公示制度，确保人民群众依法享有知情权、参与权、表决权和监督权。深入开展“五五”普法教育。完善法律援助和司法救助制度。完善村(居)务公开、民主管理和厂务公开。继续加强审计、监察监督。落实维稳工作责任制，加强社会治安综合治理，健全以群防群治力量为依托的社会治安防控体系，深入开展“打黑除恶”专项斗争，加强对治安重点地区和突出问题的整治，消除治安隐患。大力开展“平安市县”、“平安社区”、“平安村镇”、“平安单位”创建活动，推进和谐社区建设，完善社区居民自治。全面实施《信访条例》，努力把群众信访诉求解决在初始阶段和基层，妥善处理社会矛盾纠纷和群体性事件。健全公共安全防控体系，建立应急联动指挥系统，做好对突发公共卫生事件、环境污染、生产安全事故、重大自然灾害等应急处置工作，突出抓好交通、矿山、消防、食品、药品等方面的安全监管。

　　(十二)进一步加强政府自身建设，提高依法行政水平。政府肩负着组织实施经济和社会发展的职责，加强政府自身建设是实现我市经济社会加快发展的重要保证。必须进一步加强政府自身建设，着力建设为民、高效、廉洁、务实、有为政府，提高政府公信力和执行力。要进一步完善部门年度工作考核目标责任制、行政首长问责制，创新行政机关和窗口单位作风评议方式方法，继续办好“行风热线”，完善公务员评议考核制度。继续转变政府职能，强化社会管理和公共服务。坚持依法行政，开展行政执法职能的核准、界定和公告工作，健全行政执法责任制和责任追究制，严肃查处违法设置审批事项、审批不公、以审批代替监管等行为。加强行政效能监察，加快行政服务中心“一站式”电子政务行政审批电子监察系统建设和推广应用，力争年内实现并联审批。严格执行岗位责任制、首问责任制、限时办结制、服务承诺制。建立完善政府新闻发言人制度，落实重大事项决策的公开化、民主化、科学化。深入实施《肇庆市建立健全教育、制度、监督并重的惩治和预防腐败体系实施办法》，进一步落实廉政工作责任制和做好反腐败工作，严肃查处违法违纪行为，坚决纠正损害群众利益的不正之风。

　　各位代表，我们要在中共肇庆市委的正确领导下，进一步树立和落实科学发展观，同心同德，振奋精神，抢抓机遇，开拓奋进，努力完成今年的各项任务，为实现市第十次党代会和市十一届人大一次会议确定的目标任务，建设繁荣活力、文明法治、和谐安康、生态环保肇庆而努力奋斗！
